# Gene Expression Analysis of Early Stage Endometrial Cancers Reveals Unique Transcripts Associated with Grade and Histology but Not Depth of Invasion

**DOI:** 10.3389/fonc.2013.00139

**Published:** 2013-06-17

**Authors:** John I. Risinger, Jay Allard, Uma Chandran, Roger Day, Gadisetti V. R. Chandramouli, Caela Miller, Christopher Zahn, Julie Oliver, Tracy Litzi, Charlotte Marcus, Elizabeth Dubil, Kevin Byrd, Yovanni Cassablanca, Michael Becich, Andrew Berchuck, Kathleen M. Darcy, Chad A. Hamilton, Thomas P. Conrads, G. Larry Maxwell

**Affiliations:** ^1^Department of Obstetrics, Gynecology and Reproductive Biology, College of Human Medicine, Michigan State University, Grand Rapids, MI, USA; ^2^Gynecologic Oncology Service, Department of Obstetrics and Gynecology, Walter Reed National Military Medical Center, Bethesda, MD, USA; ^3^Department of Biomedical Informatics, University of Pittsburgh, Pittsburgh, PA, USA; ^4^GenEpria Consulting, Inc., Columbia, MD, USA; ^5^Department of Obstetrics and Gynecology, Uniformed Services University of the Health Sciences, Bethesda, MD, USA; ^6^Women’s Health Integrated Research Center at Inova Health System, Annandale, VA, USA; ^7^Division of Gynecologic Oncology, Duke University Medical Center, Durham, NC, USA; ^8^Department of Obstetrics and Gynecology, Inova Fairfax Hospital, Falls Church, VA, USA

**Keywords:** endometrial cancer, gene expression, stage I, RORB, IHH, DLG7, MELK

## Abstract

Endometrial cancer is the most common gynecologic malignancy in the United States but it remains poorly understood at the molecular level. This investigation was conducted to specifically assess whether gene expression changes underlie the clinical and pathologic factors traditionally used for determining treatment regimens in women with stage I endometrial cancer. These include the effect of tumor grade, depth of myometrial invasion and histotype. We utilized oligonucleotide microarrays to assess the transcript expression profile in epithelial glandular cells laser microdissected from 79 endometrioid and 12 serous stage I endometrial cancers with a heterogeneous distribution of grade and depth of myometrial invasion, along with 12 normal post-menopausal endometrial samples. Unsupervised multidimensional scaling analyses revealed that serous and endometrioid stage I cancers have similar transcript expression patterns when compared to normal controls where 900 transcripts were identified to be differentially expressed by at least fourfold (univariate *t*-test, *p* < 0.001) between the cancers and normal endometrium. This analysis also identified transcript expression differences between serous and endometrioid cancers and tumor grade, but no apparent differences were identified as a function of depth of myometrial invasion. Four genes were validated by quantitative PCR on an independent set of cancer and normal endometrium samples. These findings indicate that unique gene expression profiles are associated with histologic type and grade, but not myometrial invasion among early stage endometrial cancers. These data provide a comprehensive perspective on the molecular alterations associated with stage I endometrial cancer, particularly those subtypes that have the worst prognosis.

## Introduction

The majority of endometrial malignancies are carcinomas, which historically have been characterized as Type I or Type II on the basis of both clinical presentation as well as histopathologic variables (Deligdisch and Holinka, 1987). Based on these criteria, Type I endometrial carcinomas are usually endometrioid in histology, present with early stage disease at diagnosis, are well-differentiated with respect to grade, and are often associated with a hyper-estrogenic milieu (Berchuck and Boyd, 1995). These cancers display a high incidence of loss of function alterations in the *PTEN* tumor suppressor gene as well as defects in DNA mismatch repair resulting in microsatellite instability (Risinger et al., 1993, 1997; Tashiro et al., 1997). Endometrioid tumors may also contain activating mutations of the *CTNNB1*, *PIK3CA*, and *PIK3R1* genes, and more infrequently *KRAS2* and *FGFR2* genes (Ignar-Trowbridge et al., 1992; Fukuchi et al., 1998; Kobayashi et al., 1999; Oda et al., 2005; Hayes et al., 2006; Pollock et al., 2007). In contrast, Type II endometrial cancers usually have non-endometrioid histology, are poorly differentiated, and are frequently advanced stage at the time of diagnosis (Berchuck and Boyd, 1995). These tumors are more likely to harbor *TP53* mutation, and are characterized by widespread aneuploidy (Lukes et al., 1994; Berchuck and Boyd, 1995; Kohler et al., 1995, 1996). Type II lesions infrequently display the molecular alterations commonly associated with type I endometrioid tumors. However, the definition of Type I and Type II is imprecise particularly when high grade endometrioid cancers are considered, suggesting that more heterogeneity exists than the present dichotomous classification model (Risinger et al., 2003; Ferguson et al., 2004, 2005; Maxwell et al., 2005). Recent comprehensive genomic mutational portraits of endometrial cancer will likely aid in development of more precise classification models of uterine cancers (Kuhn et al., 2012; Le Gallo et al., 2012; Liang et al., 2012).

Transcript expression investigations by hybridization-based microarray techniques have previously demonstrated that distinct profiles are associated with different histologic types of endometrial cancer (Moreno-Bueno et al., 2003; Risinger et al., 2003; Cao et al., 2004; Ferguson et al., 2004, 2005; Maxwell et al., 2005). Although these studies revealed differentially expressed transcripts amongst endometrial cancer histotypes, there are limited data associated with these subtypes of cancer when compared to normal post-menopausal endometrial epithelium, the presumptive cell of origin for this disease. Previous studies have not been specifically designed to examine transcript expression profiles as a function of sub-stage or grade, hence a focused evaluation of early endometrial cancer and traditionally used clinicopathologic criteria is lacking (Mutter et al., 2001; Saidi et al., 2004; Wong et al., 2007). We examined the hypothesis that transcript expression profiles underlie the basic clinicopathologic variables currently used to characterize this disease. A greater understanding of distinct gene expression patterns of early endometrial carcinogenesis could result in identification of potential targets for future prognostic, therapeutic, and chemopreventive agents.

## Materials and Methods

### Tissue specimens

Stage I endometrial cancers utilized for this study were collected from patients (following counseling and written consent) that enrolled on one of two different protocols: (1) a tissue and data collection protocol approved by the Institutional Review Board (IRB) at Duke University Medical Center; and (2) a tissue and data collection protocol (GOG-136) managed by the Gynecologic Oncology Group which was approved by the IRB of each institution that provided specimens to the central repository. The current project used a de-identified sample set of specimens and data from these two resources after approvals from the Duke University IRB and the GOG Protocol Committee. Second level review was provided by the Office of Human Protections at the U.S. Medical Research and Material Command. An ethics committee review was not required to review based on the proposed research efforts.

Hematoxylin and eosin stained tissue specimens were evaluated by one of two board certified gynecologic pathologists (CZ and who else?) to confirm the original diagnosis. Only homogenous serous endometrial cancers were used for the analysis and mixed epithelial cancers were excluded. Normal endometrial samples obtained from hysterectomy specimens from 12 age-matched post-menopausal women were used for the comparison.

For the discovery analysis, samples differed by grade and stage as follows: 9 IAG1, 14 IAG2, 7 IAG3, 14 IBG1, 12 IBG2, 12 IBG3, 7 ICG1, 10 ICG2, and 6 ICG3. There were 79 endometrioid and 12 serous cancers. Sub-stage designations were based on the 1988 version of FIGO classifications of disease that more clearly describe the extent of invasion and thus allow for comparison of non-invading versus deep invading tumors as compared to the revised 2010 criteria.

Additionally, array data from six pre-menopausal endometrium samples (three proliferative and three secretory phase of the menstrual cycle) were compared to the array data from the 91 stage I cancers to assess whether ontology of differentially expressed genes varied substantially with the menopausal status of the control endometrium.

An independent set of cases used for validation of discovery gene expression data included 40 stage I endometrioid endometrial cancers, 18 stage I serous endometrial cancers, 7 normal post-menopausal endometrial samples, and 18 pre-menopausal endometrial (*n* = 9 proliferative phase and *n* = 9 secretory phase) samples.

### Tissue preparation

Laser microdissection was used to isolate cancer cells from tumors or epithelial cells from normal endometrium. Approximately 10–15 serial tissue thin sections (8 μm) for each cancer case and up to 40 serial tissue thin sections for each normal control were required to obtain sufficient RNA. Laser microdissection enabled collection of greater than 95% purity of cancer or normal epithelial cells.

### Oligonucleotide microarrays

Approximately 50 ng of total RNA was extracted from each sample and processed using the GeneChip two-cycle cDNA synthesis kit (Affymetrix Inc., Santa Clara, CA, USA). Approximately 10 μg of amplified cRNA was labeled, hybridized, washed, and scanned according to the manufacturer’s specifications (Affymetrix). The Affymetrix HG-U133 plus 2.0 GeneChip system was used to analyze over 54,000 transcripts covering 28473 UniGene clusters.

### Quantitative PCR

The expression levels of transcripts chosen for validation were determined by multiplexed PCR (TaqMan^®^ Gene Expression Assays, Applied Biosystems Inc., Foster City, CA, USA). About 18S ribosomal RNA was utilized as the reference. Samples were analyzed (Prism^®^ 7900 Sequence Detection System, ABI) according to manufacturer’s suggested protocols in triplicate. Relative expression values for each targeted transcript were calculated for each sample using the comparative *C*_T_ method. The geometric average of the mean ratios of each histologic group was calculated along with the standard error of the mean.

### Bioinformatics

The transcript expression data were normalized to a target intensity of 500 (MAS5.0, Affymetrix). Affymetrix.cel files were processed with the program GCOS to generate signal values. Multidimensional scaling (MDS) was performed using one-correlation as distance metric. Unsupervised hierarchical clustering of samples was performed with Cluster 3.0 and the dendrograms visualized with Tree View (Eisen Lab, University of California, Berkeley, CA, USA) Clustering was performed with the entire gene list (unsupervised) to determine whether there are groupings of samples and the same software was utilized to generate heat maps of the most differentially expressed genes. Supervised differential gene expression between groups of samples was performed using BRB array tools from the National Cancer Institute (http://linus.nci.nih.gov/BRB-ArrayTools.html). Parametric *p*-values are reported and the false discovery rates (FDR) were calculated using the Benjamini and Hochberg method. The identification of differentially expressed transcripts was carried out using MetaCore (GeneGo, San Diego, CA, USA). The raw data were deposited in GEO (GSE17025).

## Results

### Gene expression analysis

**Figure 1:**
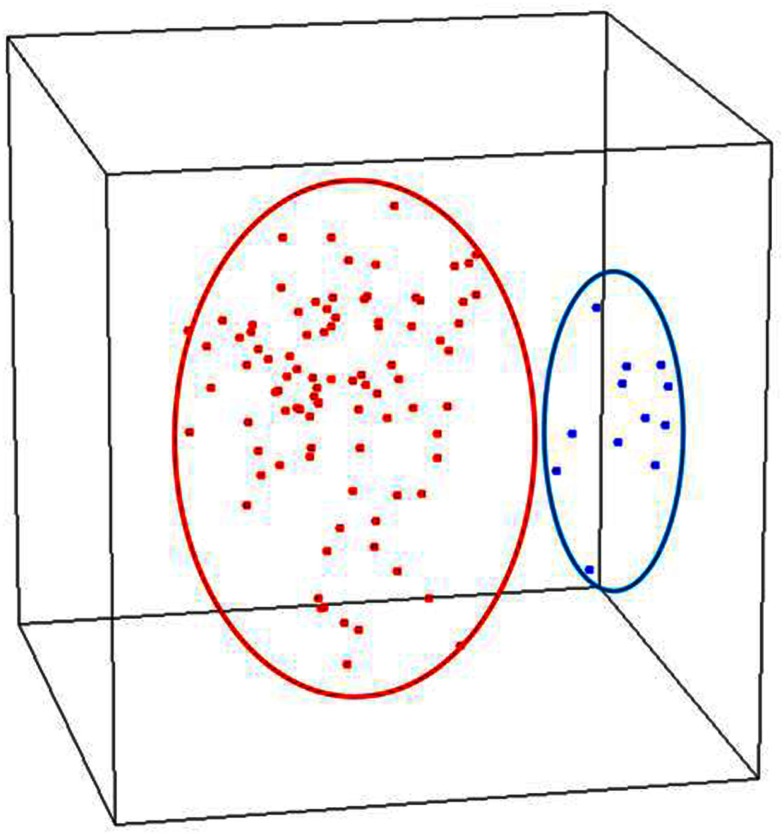
**Unsupervised analysis of stage I endometrial cancers versus normal post-menopausal endometrium controls (red, endometrial cancer; blue, normal endometrium controls)**.

We assessed the global gene expression patterns of our samples using unsupervised MDS which revealed two distinct and homogenous clusters: one comprised of normal endometrium and the other early stage endometrial cancer including both serous and endometrioid histotypes (Figure 1). When cancers were analyzed separately, the histologic subtypes appear to be distinct. We further examined these differences by identifying differentially expressed genes based on these comparisons. Specifically, we compared the 79 endometrioid endometrial cancer samples and 12 serous endometrial cancer samples to 12 normal post-menopausal endometrial tissue samples to identify statistically significant differentially regulated transcripts. This comparison of these early stage cancers with the normal endometrial controls by a univariate *t*-test revealed differential expression of 6168 transcripts (*p* < 0.001, FDR < 0.001) indicating that endometrial cancer cells possess highly distinct transcript profiles compared to glandular cells from the normal epithelium (Table S1 in Supplementary Material). We further evaluated the expression of six of these genes [Retinoic Acid Related (RAR) orphan receptor B (*RORB), PEG3, TRH, S100A8*, maternal embryonic leucine zipper kinase (*MELK), and DLG7*] using real-time quantitative PCR to validate the methodology of our array processing. Quantitative PCR results were highly consistent with the microarray analysis (Figure S1 in Supplementary Material). Specifically we included an analysis of *PEG3* and confirmed the altered expression of this transcript similarly to that we described previously (Risinger et al., 2003). These analyses indicate that our data quality was high and reproducible, thus allowing for confidence in the detailed comparisons of normal to cancer, low to high grade, non-invasive to deeply invasive and histotype of cancer that follow.

### Normal epithelium to cancer

**Figure 2:**
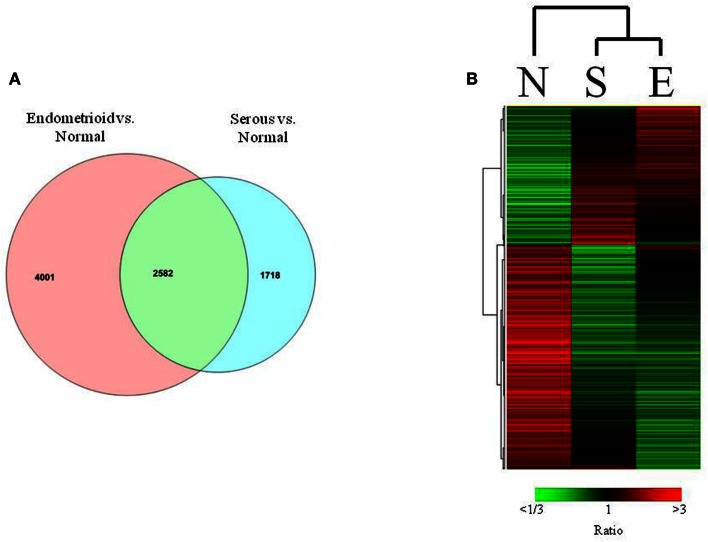
**(A)** Venn diagrams showing relationship between serous and endometrioid endometrial cancers in terms of the differentially expressed genes (*p* < 0.001) that are unique and shared among histologic subtypes when compared to normal endometrium. **(B)** Heat map of differentially expressed transcripts in matched analysis of endometrioid, serous, and normal tissues.

In the comparison of endometrioid endometrial cancers to normal endometrium, we identified 6583 transcripts that were differentially expressed (*p* < 0.001, FDR < 0.001%, Table S2 in Supplementary Material). Comparison of serous endometrial cancers versus controls revealed 4300 differentially expressed transcripts (*p* < 0.001, FDR < 0.004%) between the two groups (Figure 2A and Table S3 in Supplementary Material).

Although these two histotypes of endometrial cancer have many transcripts that are differentially expressed compared to normal controls, they also may share a significant proportion of differentially expressed transcripts as previously reported (Risinger et al., 2003). In this analysis, 2582 differentially expressed transcripts were found in association with both histology subtypes compared to control, with 432 of these transcripts demonstrating fourfold or greater expression differences.

Given the unbalanced number of endometrioid endometrial cancers in this sample set, we further refined our comparisons to normal by selecting a set of 12 endometrioid endometrial cancers closely matched by sub-stage and grade to the serous endometrial cancers. Using these balanced sets of cases, the comparison of endometrioid cancer to normal identified about 5500 differentially expressed transcripts (*p* < 0.001). A comparison of the differential expressions between the histotypes assessed by matched sample sizes reveals common and distinct transcripts (Figure 2B). Furthermore, when these data were analyzed by MDS all three types of samples were distinctly clustered confirming the validity of gross differences between the two histotypes irrespective of the large number of endometrioid cases. Because these selected endometrioid cases were matched to serous in terms of grade it also strongly suggests that these distinctions are maintained even between high grade endometrioid and serous carcinoma.

### Transcript expression and tumor grade

To understand if distinct transcript expression changes associated with tumor grade exist, an unsupervised MDS analysis of grade in the endometrial cancer samples was performed to identify changes in transcript expression associated with poorly differentiated tumors. When the entire sample set was evaluated, including both serous and endometrioid cancers, the grade three endometrioid cancers did not appear to segregate with the serous tumors (grade 3 by definition) Because serous cancers were shown to be different compared to endometrioid tumors we conducted a focused analysis of transcript expression levels as a function of grade in endometrioid endometrial cancer (grade 1, *n* = 30 versus grade 3, *n* = 13). Although an unsupervised MDS analysis indicated no distinct clustering according to grade (data not shown), a supervised differential expression analysis resulted in identification of 498 statistically significant (*p* < 0.001, FDR < 0.1) differentially abundant transcripts (Tables S1 and S4 in Supplementary Material).

### Effect of myometrial invasion

To address whether depth of invasion is the result of biologic differences between cancers or simply a reflection of the temporal progression of disease, we analyzed the gene expression of endometrioid cases with no myometrial invasion (*n* = 30) against those that were deeply invasive (*n* = 23). In 2010, FIGO updated endometrial cancer staging to reflect in part the relative lack of prognostic difference between the previous stage IA and stage IB sub stages using 1988 FIGO staging criteria. Despite these clinical data, we chose to compare only the extremes of invasion (superficial IA to deeply invasive IC). Unsupervised MDS analysis indicated no separation of stage IA cancers versus stage IC cases (data not shown) suggesting that depth of invasion is not reflected in a global transcript expression pattern. Univariate *t*-tests indicated only 46 transcripts (*p* < 0.001, FDR 0.9) which may be found merely by random chance.

### Validation of selected genes in independent sample sets

**Figure 3:**
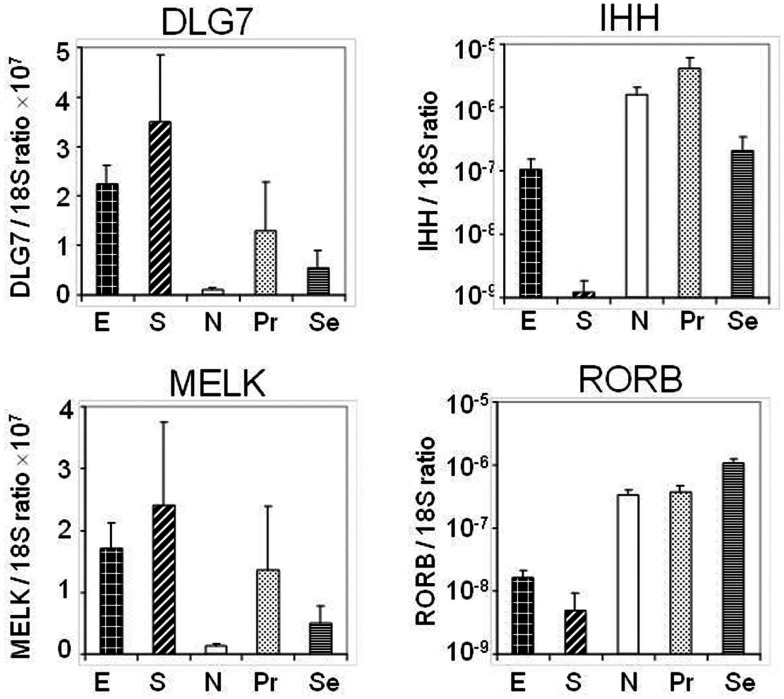
**Quantitative PCR of four selected transcripts performed on an independent set of endometrial tissues to include stage I serous (S) and endometrioid (E) cancers, normal post menopausal epithelium (N), proliferative epithelium (Pr), and secretory epithelium (Se)**.

**Figure 4:**
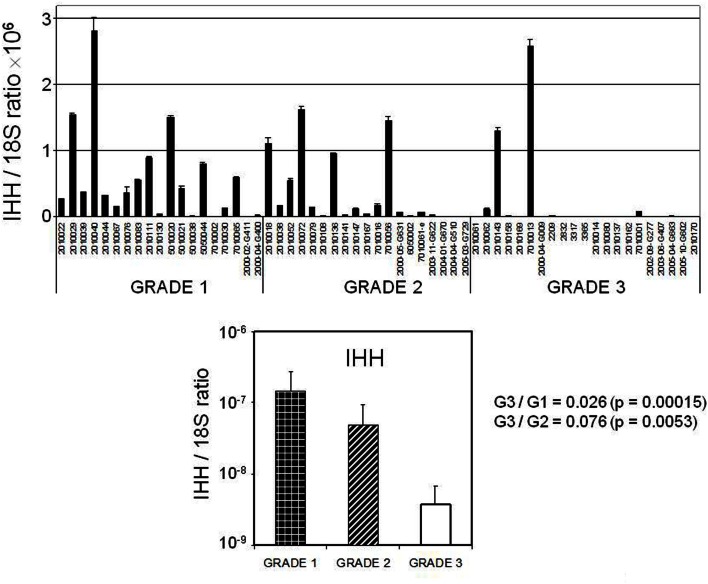
**Expression of *IHH* in all endometrial cancer samples according to tumor grade**.

Four transcripts [*RORB*, Indian Hedgehog gene (*IHH*), *DLG7* (*DLGAP5*), and *MELK*] whose expression levels were identified as altered by hybridization-based microarray analysis in the discovery set were chosen for validation in an external sample set comprised of 58 endometrial cancer (40 endometrioid and 18 serous) and 25 normal endometrial (7 post-menopausal and 18 pre-menopausal) tissue specimens utilizing by qRT-PCR (Figure 3). Specifically *RORB* expression was highly down-regulated in cancers including both serous and endometrioid types. *DLG7* and *MELK* were up-regulated in cancers compared to normal endometrial specimens and more highly in serous cancer as compared to endometrioid cancer. We also confirmed the serous cancer-specific down-regulation of *IHH*. Expression of *IHH* and its association with tumor grade is shown in Figure 4.

### Biological classification of differentially expressed genes

**Figure 5:**
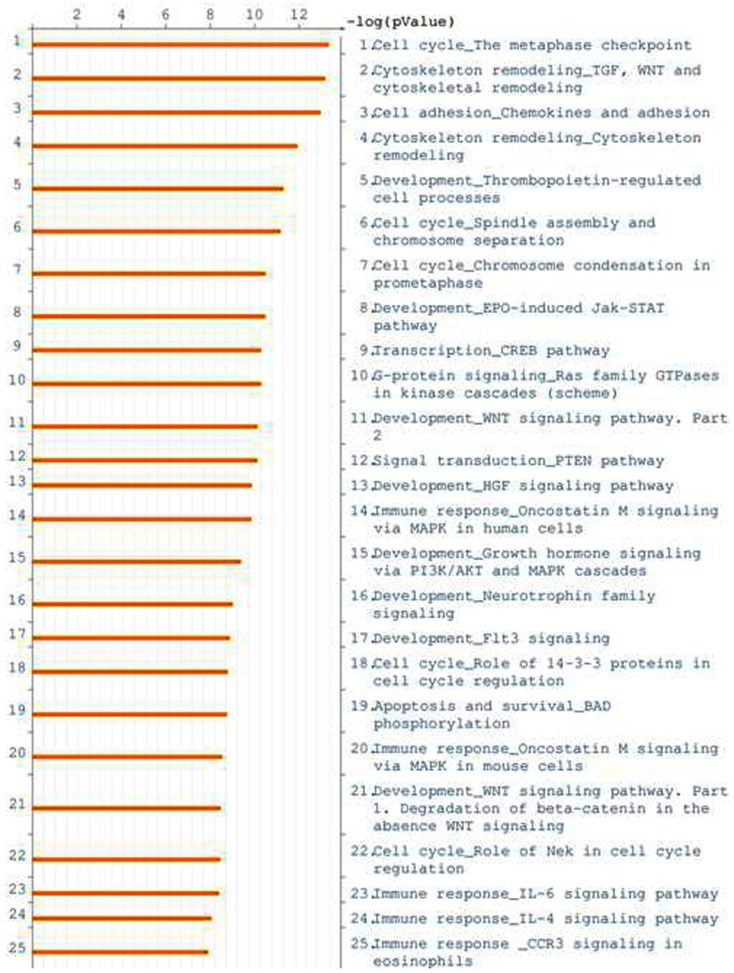
**Genes differentially expressed in early stage endometrial samples compared to normal epithelia were evaluated for enrichment of biologic pathways using the MetaCore (GeneGo) analysis tool**. A histogram of log *p*-values is shown; the list is arranged in descending order with the most significant pathways at the top.

We investigated whether certain cellular pathways are dysregulated in early stage cancer. A comparison of the endometrial cancers (serous and endometrioid) versus normal post-menopausal endometrium control gene list using the MetaCore (GeneGo) pathway and ontology mining database revealed enrichment of pathways involved in cell cycle, cytoskeletal remodeling, chemokines in cell adhesion, and several signaling pathways including PTEN, Wnt, Flt, and CREB (Figure 5). In order to determine if the transcripts identified in this study were mostly reflective of a “proliferation signature of cancer,” we also evaluated array data from a comparison of 91 stage I cancers to laser microdissected epithelium from a separate set of 6 pre-menopausal women (Table S5 in Supplementary Material). Comparisons of ontology results between the cancers versus post-menopausal controls and the cancers versus pre-menopausal controls revealed that cell cycle and cell division ontologies were still the most significant ontologies resultant from pathway analysis of the differentially expressed genes (data not shown).

## Discussion

In this study, we examined the transcript expression profiles of stage I endometrial cancers and post-menopausal epithelium to gain insight into the biology and to determine the relevance of expression profile differences to clinical and pathologic variables used to characterize this disease. Although several investigations have previously examined gene expression profiles associated with different subtypes of endometrial cancer, few have been designed specifically to identify transcripts that are differentially expressed between endometrial cancer and normal endometrium, and none have examined the effect of sub-stage on transcript expression. We selected a heterogeneous group of stage I endometrial cancers on the basis of grade and degree of myometrial invasion representing the full range of stage I sub-strata.

Two previous study performed comparisons of normal post-menopausal samples with serous and endometrioid cancers (Risinger et al., 2003). Our previous study consisted of only 7 normal specimens compared to 39 endometrial cancers (including 6 serous and 11 endometrioid stage I cancers) limiting the power of performing stratified analyses. Similarly the only other large study of microdissected normal endometrium and endometrial cancer did not analyze their data based on stage I sub-stage criteria nor are these data publicly available to perform such comparisons (Wong et al., 2007).

The present study of endometrial cancer gene expression differed from most prior studies in the selection of normal control specimens (Mutter et al., 2001; Saidi et al., 2004; Wong et al., 2007). We chose to examine normal endometria exclusively from post-menopausal women since the vast majority of endometrioid and almost all serous endometrial cancers occur after menopause. Although we did not have clinical data to reflect which of these women might have been on hormone replacement therapy, each normal endometrial specimen was reviewed prior to inclusion to confirm an atrophic post-menopausal pattern. The choice of what constitutes a “normal control” for endometrial cancer is problematic. While serous cancers are thought to develop from atrophic endometrium, some endometrioid cancers may occur in obese individuals who may have a post-menopausal endometrium that is stimulated in part by their weight-related estrogenic milieu. About 20% of women develop endometrial cancer prior to menopause. Approximately half of the endometrial cancers in this study were from post-menopausal women who were considered to be normal weight. Our choice of age matched, yet atrophic, endometrium might be postulated to enhance the discovery of transcripts related to cell division. Analysis of Gene Ontology (GO) does indeed identify cell proliferation and apoptosis as some of the most important ontologies (Figure 5). To determine if the transcripts identified in this study were mostly reflective of a “proliferation signature,” we also compared these same stage I cancers to laser microdissected epithelium from a separate set of array data obtained from pre-menopausal women. Even in this comparison, cell cycle and cell division are the most significant ontologies resultant from pathway analysis of significant genes. This additional comparison suggests that gene expression changes related to these processes in cancer predominates over the menopausal status of the normal control. Although serous and endometrioid stage I endometrial cancers share many differentially expressed genes when compared to normal controls, they are distinctly different from one another, a finding we and others have previously reported (Risinger et al., 2003; Maxwell et al., 2005; Zorn et al., 2005). These previous analyses did not include significant numbers of stage I serous cancers to determine whether these are a distinct subtype. In the set of transcripts that we sought to validate, we specifically selected some molecules that were significantly different among endometrioid and serous carcinomas compared to normal endometrium. Among these, we found that two previously implicated stem cell associated markers, the disks large homolog 7 (*DLG7*, also known as *DLGAP5)* and the maternal embryonic leucine zipper kinase *MELK* transcripts were elevated in endometrial cancer specimens compared to controls. Recently *MELK* has been described as over-expressed in carcinomas and in particular their stem cell niche (Risinger et al., 2003; Liu et al., 2006; Marie et al., 2008; Nakano et al., 2008; Pickard et al., 2009). The exact function of the *MELK* gene product (MELK) in normal and tumor biology remains to be determined. The identification of up-regulated *MELK* has the potential to be exploited clinically if a tumor growth phenotype is attributed to MELK, as kinases have often been effectively targeted by small molecule therapy in cancer. Similarly the *DLG7* gene product (DLG7) is associated with several malignancies (Chiu et al., 2002; Tsou et al., 2003; Gudmundsson et al., 2007). Furthermore DLG7 may be directly involved in cell transformation (Yu et al., 2005). Given that over-expression of these two genes was more prominent in serous versus endometrioid cancers, our findings could suggest that the resistance of serous cancers to contemporary therapy may be in part due to an increased prominence of tumor cell progenitors within these cancers or reflect a more aggressive proliferative lesion. Based on these findings, further investigation is warranted in identifying the functions of these two genes in endometrial cancer.

Among the validated differentially expressed transcripts, the *IHH* was noted to be highly down-regulated in serous cancers. The *IHH* gene product (IHH) has been extensively studied in the uterus and is a key signaling molecule in regulation of the uterine epithelium in preparation for implantation (Lee et al., 2006; Franco et al., 2010). Furthermore, *IHH* expression is regulated by ovarian steroids, and signaling occurs through progestin and surrounding stroma (Simon et al., 2009). Our validation data also revealed the significant up-regulation of *IHH* in the proliferative endometrial epithelium consistent with other mRNA studies on normal uterine samples sampled throughout the menstrual phase (Talbi et al., 2006). Decreased *IHH* expression has also been described in endometriosis (Smith et al., 2011). Our array discovery- and qRT-PCR-based validation data indicated a stark reduction of expression in the serous cancers and in some endometrioid cancers. Importantly we also noted the presence of *IHH* on our list of differentially expressed transcripts related to tumor grade. Specifically *IHH* was distinctly down-regulated in most high grade cancers regardless of histotype (Figure 4). Poorly differentiated endometrial cancers are more frequently associated with lack of expression of the estrogen and progesterone receptor. We further explored this by examining *PGR* mRNA levels in these cancers and found that loss of *PGR* and *IHH* expression were correlated. Despite its importance in normal uterine biology, the Hedgehog pathway has not been extensively investigated in uterine carcinoma. Given the role of IHH in negatively regulating the Hedgehog signaling pathway, these data suggest that some high grade endometrial cancers might benefit from targeted Hedgehog pathway intervention.

Down-regulation of the nuclear orphan receptor *RORB*, a receptor that is part of the NR1 nuclear receptor family, was one of the most strikingly down-regulated transcripts in both serous and endometrioid cancer. We noted robust expression in both pre- and post-menopausal endometrium controls. Since expression was markedly higher in secretory compared to proliferative endometrium, the loss of *RORB* expression may be related to endometrial differentiation, a process known to be mediated in part by retinoic acid (a ligand for RORB) (Stehlin-Gaon et al., 2003). Retinoic acid has a long known function in modulating steroid driven proliferation and differentiation responses in the uterus as well as modulating carcinogenesis (Siddiqui et al., 1994; Loughney et al., 1995; Brar et al., 1996; Li et al., 2002, 2005; Cheng et al., 2011). The loss of *RORB* expression in endometrial cancer warrants further investigation.

In the comparing normal post-menopausal to cancer subtypes, we noted differentially expressed transcripts common to both serous and endometrioid endometrial cancers, many of which could be considered as targets for preventive or therapeutic regimens (Tables S2 and S3 in Supplementary Material). For example, the *TPX2* gene encodes an Aurora kinase A-stimulated microtubule regulating molecule that when over-expressed in cancer is associated with tumorigenesis (Scharer et al., 2008; Warner et al., 2009). In addition, specific targeting of *TPX2* with short interfering RNAs in pancreas cancer cells results in decreased proliferation and tumorigenicity. Cancer cells in which TPX2 levels have been suppressed by small interfering RNAs are more sensitive to the microtubule stabilizing cancer drug paclitaxel (Warner et al., 2009). Similarly, disrupting Aurora kinase activity sensitizes ovary cancer cells to paclitaxel (Scharer et al., 2008). Both DLGAP5 and TPX2 appear to rely on increased Aurora kinase function. Estrogen induced tumors in hamsters overexpressing Aurora kinase develop uterine like stem cell tumors in their kidneys. It is possible that uncontrolled estrogenic stimulus in endometrial cells is mediated in part through Aurora kinases which are active in endometrial cancers (Kurai et al., 2005). Although many stage I endometrial cancers are cured by surgery alone, those that recur have very poor prognosis with limited treatment options. Investigation of Aurora kinases and/or TPX2 targeted therapies should be considered in “high risk” early stage cancers.

The loss of cell polarity and normal cell adhesion processes are central features of cellular transformation and metastasis. We noted a distinct enrichment for dysregulated genes in the cell adhesion (tetraspanins and integrin) pathway by GeneGo analysis of all of the differentially expressed genes. Although members of the integrins and tetraspanins have been examined in endometrial cancer there has not been focused study for many of these key molecules in early disease. Our data show that signaling through deregulated integrin and tetraspanins may account for the observed increased expression of focal adhesion kinase (FAK) and ezrin in this endometrial cancer data set. FAK is a central component regulating invasion and metastasis and limited data suggest it is up-regulated at the protein level in some endometrial carcinomas (Livasy et al., 2004). Similarly, increased expression of the cytoskeletal linking protein ezrin is seen in endometrial hyperplasia and carcinoma (Ohtani et al., 1999, 2002; Kobel et al., 2006). In stage I endometrial cancers, ezrin expression was also linked to poorer prognosis (Kobel et al., 2006). Our network data suggests additional linkages related to these signaling pathways and is depicted in Figure S1 in Supplementary Material.

Our data showed the absence of statistically significant differentially expressed genes associated with deep myometrial invasion, a poor prognostic factor associated with an increased risk of extra-uterine metastasis. A dogmatic theme in molecular carcinogenesis is the progressive accumulation of genetic defects as cells progress from normal cells to premalignant cells to carcinomas. Most interestingly our study found few genes with expression changes of statistical significance between non-invading and deeply invaded stage I endometrial cancers. These data suggest that a large component of endometrial cancer progression may be temporal and not due to further accumulation of cellular defects reflective in gene expression. LKB1 previously implicated in endometrial cancer invasion (26), was not among the few differentially expressed genes identified.

In summary, we determined that stage I endometrial cancers have unique global expression patterns based on their histologic subtype. However, this study importantly describes a large number of gene transcripts that are distinctly dysregulated regardless of histotype when these cancers are compared to normal endometrial epithelium. Most surprising was the distinct lack of changes that existed between superficially invasive stage I cancers when compared to those that are deeply invasive, which suggests that depth of invasion may have a temporal etiology and does not necessarily reflect a unique biological expression profile that develops as an endometrial cancer becomes more advanced. Future studies aimed at identifying effective prognostics for predicting stage I cancer recurrence will likely have to focus away from the traditional clinical and pathologic criteria in order to be effective. Given that many early stage endometrial cancers share significant common gene expression changes, broad-based preventive and chemotherapeutic strategies have the potential to be developed that impact both endometrioid and serous subtypes of endometrial carcinoma.

## Conflict of Interest Statement

The authors declare that the research was conducted in the absence of any commercial or financial relationships that could be construed as a potential conflict of interest.

## Supplementary Material

The Supplementary Material for this article can be found online at http://www.frontiersin.org/Women’s_Cancer/10.3389/fonc.2013.00139/abstract

Supplementary Figure S1 **Transcript expression from quantitative PCR (top row) and microarray analysis (bottom row) for six selected genes (RORB, PEG3, TRH, S100A8, MELK, and DLG7) differentially expressed between endometrial carcinoma endometrioid (E), papillary serous carcinoma (PS), and normal endometrium (N)**.
