# Supplementary figures and images for: Gene Expression Analysis of Early Stage Endometrial Cancers Reveals Unique Transcripts Associated with Grade and Histology but Not Depth of Invasion
Source: Front Oncol. 2013 Jun 17;3:139. doi: 10.3389/fonc.2013.00139 (PMC3683664; doi:10.3389/fonc.2013.00139)

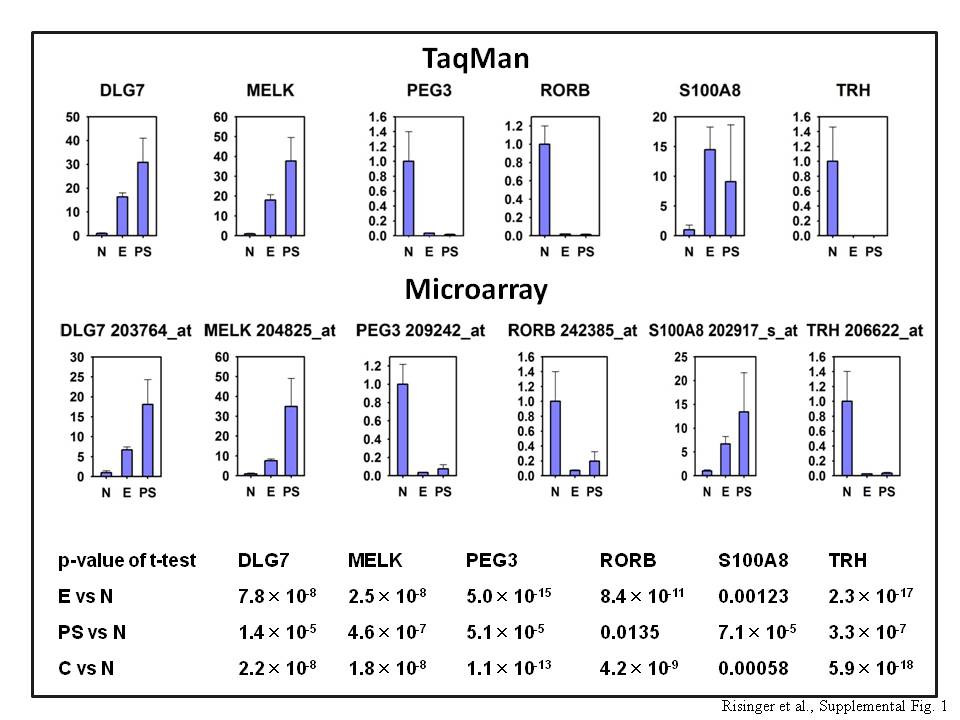

Supplement: Supplementary Figure S1 — Transcript expression from quantitative PCR (top row) and microarray analysis (bottom row) for six selected genes (RORB, PEG3, TRH, S100A8, MELK, and DLG7) differentially expressed between endometrial carcinoma endometrioid (E), papillary serous carcinoma (PS), and normal endometrium (N). [file Figure-S1.JPEG]
